# Exoskeleton-assisted training to enhance lower limb motor recovery in subacute stroke: does timing matter? A pilot randomized trial

**DOI:** 10.3389/fstro.2024.1379083

**Published:** 2024-05-14

**Authors:** Jonas Schröder, Laetitia Yperzeele, Elissa Embrechts, Renata Loureiro-Chaves, Ann Hallemans, Christophe Lafosse, Steven Truijen, Gert Kwakkel, Wim Saeys

**Affiliations:** ^1^Research Group MOVANT, Department of Rehabilitation Sciences and Physiotherapy, University of Antwerp, Antwerp, Belgium; ^2^Neurovascular Center Antwerp and Stroke Unit, Department of Neurology, Antwerp University Hospital, Edegem, Belgium; ^3^Research Group on Translational Neurosciences, University of Antwerp, Antwerp, Belgium; ^4^RevArte Rehabilitation Hospital, Edegem, Belgium; ^5^Department of Rehabilitation Medicine, Amsterdam Movement Sciences-Rehabilitation and Development, Amsterdam Neurosciences-Neurovascular Disorders, Amsterdam UMC, Location Vrije Universiteit Amsterdam, Amsterdam, Netherlands; ^6^Department of Physical Therapy and Human Movement Sciences, Northwestern University, Chicago, IL, United States; ^7^Department of Neurorehabilitation, Amsterdam Rehabilitation Research Centre, Reade, Amsterdam, Netherlands

**Keywords:** stroke, lower extremity, postural balance, walking, robotics, clinical trial, exoskeleton

## Abstract

**Background:**

Lower limb motor recovery, including abnormal muscle synergies, occurs mainly within the first 5–8 weeks after a stroke. This suggests the importance of delivering impairment-focused therapies, such as therapeutic robots that promote symmetric gait, during this time-sensitive period, following the principle of “the earlier, the better.”

**Objective:**

First, to compare early robotic training (ERT) with usual care (UC) against UC alone on restoring intralimb muscle synergies and interlimb symmetry during functional tasks; Second, to investigate whether ERT is superior to delayed robotic training (DRT) starting after the proposed time-sensitive period.

**Methods:**

This observer-blinded, randomized pilot trial with crossover design involved 19 nonambulatory adults included within 14 days poststroke. Those allocated to ERT (*N* = 10) received immediately 4 weeks of training (16 sessions, 4× /week) with the Ekso GT^®^ above UC and were compared with the DRT group (*N* = 9) who received UC alone at this point. Thereafter a 3-week UC period followed to investigate sustainability of ERT and the interventional roles were exchanged; at about week 8 poststroke DRT subjects started the same experimental robotic protocol and ERT subjects continued UC as controls. Outcomes included changes in Fugl-Meyer lower extremity scores (FM-LE) reflecting muscle synergies, weight-bearing asymmetry (WBA), and dynamic control asymmetry (DCA) during quiet standing. Functional ambulation category (FAC) was used to classify walking independence (cut-off ≥4).

**Results:**

A trend toward earlier reacquisition of walking independence favoring ERT with UC over UC was not accompanied by differences in FM-LE, WBA, or DCA (first objective). Thereafter, DRT with UC did not yield any significant changes relative to UC, such that no between-group differences were found favoring restorative effects of ERT over DRT (second objective).

**Conclusion:**

This pilot trial shows the feasibility of investigating a wearable exoskeleton as an adjunct therapy in subacute stroke. Nevertheless, our preliminary findings suggest that motor recovery of lower limb muscle synergies was not enhanced by 4 weeks of robotic training to reduce compensations with the less-affected side, irrespective of the timing of application.

**Trial registration:**

ClinicalTrials.gov, identifier: NCT03727919.

## Introduction

Approximately 65%−80% of stroke survivors eventually regain the ability to walk independently within the first 3–6 months poststroke (Jorgensen et al., 1995; Veerbeek et al., 2011; Kennedy et al., 2021). However, spontaneous neurological recovery from motor impairments affecting the lower limb (e.g., abnormal muscle synergies) seems to plateau within 5–8 weeks poststroke, which parallels recovery courses observed for the paretic upper limb (Duncan et al., 1994; Kwakkel et al., 2006; Schroder et al., 2023). In most cases, motor recovery is incomplete and synergistic muscular co-activation persists when performing functional tasks as standing and walking (Garland et al., 2007; Buurke et al., 2008). As a consequence, people with stroke typically prefer asymmetric postures (Laufer et al., 2003; Garland et al., 2007; Roerdink et al., 2009) and stepping patterns (Kwakkel and Wagenaar, 2002; Patterson et al., 2015) to regain independence by compensating with the less-affected limb. The critical recovery period is associated with enhanced levels of neuroplasticity (Murphy and Corbett, 2009; Zeiler, 2019). This suggests an ideal time to deliver impairment-focused rehabilitation therapies. Therefore, the question arises as to whether patients who are unable to walk at onset can be trained to enhance lower limb motor recovery and promote a normal, symmetrical gait if motor training is delivered in a timely manner according to the principle “the earlier, the better.”

Exoskeleton-type robots may be an ideal therapeutic tool for addressing the abovementioned question. These devices are designed to provide more task-specific practice (Louie and Eng, 2016; Schroder et al., 2019), which is an important requirement for improving walking (Veerbeek et al., 2014; Hornby, 2022), while also “normalizing” hemiparetic gait by mimicking the symmetrical step trajectories of able-bodied controls (Hidler et al., 2008; van Kammen et al., 2017). However, despite a trend favoring robotic training as an adjunct therapy to usual care (UC) for achieving walking independence within the first 3 months poststroke (Schroder et al., 2019; Mehrholz et al., 2020), hardly any exoskeleton trials completed their intervention within the critical recovery period of the first 5–8 weeks poststroke. Moreover, trials often lack objective biomechanical outcomes that reflect quality of movement (Nedergard et al., 2021), such as re-emergence of interlimb symmetry in center-of-pressure measures reflecting balance control (Roerdink et al., 2009; Roelofs et al., 2018) or spatiotemporal stepping parameters (Patterson et al., 2010, 2015) as hallmark features of a normative bipedal gait. The first, second, and third Stroke Recovery and Rehabilitation Roundtables (SRRR) of the International Stroke Recovery and Rehabilitation Alliance^1^ (Bernhardt et al., 2017; Kwakkel et al., 2017, 2019; Van Criekinge et al., 2024) recommend applying these so-called performance assays in trials to distinguish task improvements achieved by behavioral restitution from compensation, thereby contributing to our understanding of interaction effects between spontaneous and learning-dependent recovery induced by early-delivered therapies.

Acknowledging the lack of early-starting exoskeleton trials, this pilot study was conducted. In addition to exploring the feasibility of using the Ekso GT^®^ wearable exoskeleton for overground training (Louie and Eng, 2016; Louie et al., 2020, 2021) in a primary inpatient rehabilitation setting, our aim was to investigate preliminary effects of a 4-week early robotic training (ERT) intervention with UC against UC alone. Regarding our first objective, we hypothesized that ERT as an adjunct therapy improves muscle synergies, as reflected by changes in Fugl-Meyer Lower Extremity scores (FM-LE) that exceed those observed in the controls. Due to significant impairment reductions, we further expected more equal limb contributions during tasks in terms of weight-bearing asymmetry (WBA) and dynamic control asymmetry (DCA) while quiet standing and stepping asymmetries while walking.

Similar to the recent CPASS (Critical Period After Stroke Study) trial by Dromerick et al. (2015, 2021) suggesting a critical poststroke window for arm-hand skill training, our second aim was to investigate whether the delivery of lower limb robotic training at different time points in the subacute phase matters to achieve behavioral restitution. Therefore, we investigated whether training effects on restoring lower limb muscle synergies to promote symmetry are pronounced when applied in the first 5 weeks poststroke relative to delayed robotic training (DRT) delivered above UC at 8 weeks poststroke. Regarding our second objective, which concerns timing, we hypothesized that DRT with UC would be less effective in improving FM-LE scores and, thereby, restore symmetry to improve functional tasks than ERT with UC in the early recovery period. UC alone was used as the comparator during both timings.

## Materials and methods

This study is part of the TARGEt research project (Temporal Analyses of the Responsiveness of hemiplegic Gait and standing balance Early poststroke), funded by the Research Foundation Flanders (FWO), Belgium. The study protocol was approved by the medical ethics committee of the University Hospital Antwerp and UAntwerpen (No. 18/25/305; Belgium trial registration no. B300201837010) and registered online (ClinicalTrials.gov, no. NCT03727919). The findings are reported according to CONSORT guidelines adapted for pilot trials (Eldridge et al., 2016).

### Patient selection

Adults referred to the neurology wards of the Antwerp University Hospital (Edegem, BE) and GZA hospitals St Augustinus (Wilrijk, BE) and St Vincentius (Antwerp, BE) with suspicion of stroke were screened. Potential candidates were identified as being 18–90 years old, having a first-ever, CT- or MRI-confirmed cortical, subcortical, or midbrain infarct or hemorrhage, and exhibiting one-sided leg weakness (i.e., hemiplegia).

Information about the study was presented to each potential candidate. Once informed consent was given, eligibility was confirmed if participants required inpatient rehabilitation, could be included between 5 and 14 days poststroke to start early interventions, exhibited persistent leg weakness [i.e., Motricity Index lower extremity score (MI-LE) ≤ 75] and walking dependency [i.e., Functional Ambulation Category (FAC) ≤ 1] at the time of inclusion, and had no other significant orthopedic or neurological condition or any contraindications for using the exoskeleton, e.g., body weight >95 kg, severe lower limb hypertonia/contracture.

### Design

**Figure 1:**
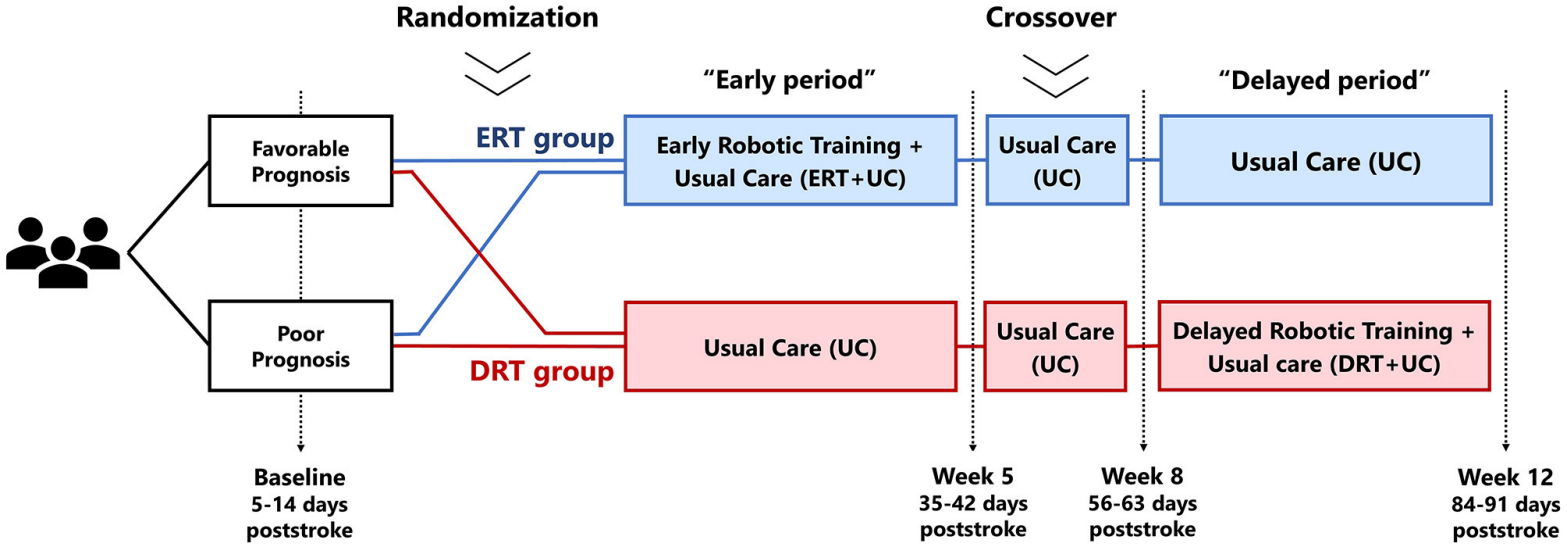
Design of the clinical trial. ERT, early robotic training; DRT, delayed robotic training; UC, usual care.

The present study had an observer-blinded, randomized crossover design (Figure 1). At the baseline measurement (i.e., 5–14 days poststroke), participants were allocated to either the ERT or DRT study arms. Randomization was concealed by using sealed opaque envelopes and executed by an uninvolved person. Randomization was blocked (2-by-2 ratio) and stratified according to the prognosis for achieving walking independence (i.e., favorable FAC ≥4 or poor FAC < 4). Following the validated EPOS (Early Prognosis of functional Outcome after Stroke) model (Veerbeek et al., 2011, 2022), a favorable prognosis was defined as having sitting balance (i.e., Trunk Control Test sitting item >25) and leg strength (i.e., MI-LE ≥25). A poor prognosis was assigned if either of the determinants were more impaired.

The experimental intervention was a 4-week robotic training program delivered additional to UC. The ERT arm received this intervention immediately after inclusion and up to the fifth week poststroke, whereas the DRT group received UC as controls. After a 3-week period consisting of UC alone to investigate the sustainability of ERT, the intervention roles were exchanged, such that the DRT group received the same experimental protocol between weeks 8 and 12 poststroke, whereas the ERT participants continued UC as controls. The delayed timing was consciously set as a control reference to deliver the same intervention approximately after the critical recovery period while maintaining within the boundaries of inpatient hospitalization. This is about 3–4 months in those with significant gait limitations, as shown by previous trials in our facilities (Saeys et al., 2012; Van Criekinge et al., 2020). Thus, between-group comparisons for estimating treatment effects were ERT with UC vs. UC in the early period, and DRT with UC vs. UC in the delayed period.

Measurements were performed at baseline and at weeks 5, 8, and 12 poststroke. A trained, blinded assessor (EE or RLC) rated subjective clinical scales in a specific subject during face-to-face sessions. Objective biomechanical evaluations were obtained using computerized laboratory devices, operated by the study coordinator (JS) who was aware of treatment allocation. Standing balance evaluations started once participants could bipedal stand [i.e., Berg Balance Scale unsupported standing item (BBS-stand) ≥2]. Walking evaluations started once participants could walk under supervision (i.e., FAC ≥3).

### Intervention

#### Robotic training

We used the Ekso GT^®^ (Ekso Bionics, CA, US) wearable exoskeleton (Louie and Eng, 2016; Louie et al., 2020, 2021), consisting of motorized limbs that provide bilateral hip and knee motion in the sagittal plane to practice overground walking. A passive spring-loaded joint maintained the ankles in a neutral position via footplates to assist foot clearance. The device must be prepared to fit the patients' body measurements, including hip width and upper and lower leg length.

The motorized legs can be moved with full or partial assistance to encourage active patient involvement and progress training difficulty. Stepping automaticity was always set to “ProStep,” such that a step was triggered when the opposite limb was sufficiently weight-loaded. In other words, the patient must lean left and forward to signal the exoskeleton to right step, and vice versa. The threshold of loading was adjusted in the system's software to encourage symmetric weight shifting, particularly toward the paretic leg, while avoiding “over-leaning.” Spatiotemporal variables, such as step height and length or swing speed, were adjusted to ensure a comfortable symmetrical gait pattern with progression by increasing, for example, step lengths and speed. The degree of ankle stiffness was lowered if voluntary ankle strength improved. Within these global guidelines, therapists were free to individualize settings to guarantee safety and improve training efficacy according to their expertise.

Each session lasted ~45 min to provide sufficient practice time, in addition to preparation and resting breaks, and was delivered by a single licensed therapist daily, 4 × /week. Practice started with establishing a symmetric stance with equal weight-bearing while wearing the exoskeleton and progressed toward stepping. Eventually, the goal was to achieve per session ~20 min time on task and ~1,000 steps. We did not use aids (e.g., cane) to facilitate a normative walking pattern.

#### Usual care

UC was not modulated and was provided continuously until discharge. Although UC was not standardized, it typically consisted of daily 60-min sessions of physiotherapy and occupational therapy, 5 × /week, besides nursing care. In general, physiotherapy targeted voluntary movement control and independent transferring or walking following the Bobath concept, and occupational therapy focused on upper limb activities such as dressing or eating. Additional speech or cognitive therapy was provided as needed.

### Outcomes

#### Intervention characteristics

The time on task (upright position and stepping) and the steps count were recorded by the robot. Intervention-related adverse events and negative side effects were monitored by the therapist during and after each session.

#### Clinical outcomes

FM-LE (0–34) was used to measure muscle synergies. FM-LE is a widely used valid and reliable measure of poststroke motor impairment (Gladstone et al., 2002; Sullivan et al., 2011; Van Criekinge et al., 2024). Increasing scores reflect improved dissociation of willed movement from abnormal synergistic co-activation. BBS-stand (0–4) measures the ability to bipedal stand. FAC (0–5) measures the ability to walk without support or supervision. BBS-stand and FAC are ordinal scales and were dichotomized to categorize independence at cut-offs BBS-stand ≥2 and FAC ≥4, respectively.

#### Biomechanical outcomes

Biomechanics samples were collected at the M^2^OCEAN laboratory of the UAntwerp, following standardized protocols for measuring quiet standing balance (Schroder et al., 2022, 2023) and walking (Van Criekinge et al., 2017, 2020). Data analysis was performed using custom-written MATLAB (version 2018a) algorithms.

Balance was evaluated during quiet bipedal standing for 40 s while placing one foot each on a force platform (type OR 6–7, AMTI, MA, USA). Data were low-pass filtered (Butterworth 2nd order, cut-off 12.5 Hz). The first 10 s were removed, and three trials were averaged to maximize reliability (Ruhe et al., 2011). The root mean square of the center-of-pressure velocities at the limbs combined was calculated as a measure of anteroposterior (COPvel-ap) and mediolateral (COPvel-ml) postural stability (Roelofs et al., 2018). WBA is calculated as the percentage weight on the less-affected side minus 50%. DCA reflects each limb's balance control contribution as a symmetric index of the individual-limb COPvel-ap (Roelofs et al., 2018). With respect to WBA and DCA, 0% indicates perfect symmetry, and positive values reflect a larger contribution by the less-affected limb. Because COP signals are sensitive to errors when applied forces are low, DCA was set arbitrarily to 160% (i.e., largest asymmetry recorded) if < 20% body weight was recorded on the less-affected limb.

Motion capture (VICON Motion Systems Ltd, Oxford, UK) was used to evaluate step trajectories during barefoot walking at comfortable speeds over a 10-m walkway. Foot markers (heel, ankle, 2nd toe) were labeled and low-pass filtered (Butterworth 2nd order, cut-off 6 Hz). Foot-strike and foot-off events were determined using a coordinate-based algorithm (Zeni et al., 2008) for at least eight strides in the walkway center. We calculated step lengths (i.e., difference in ankle position between foot-strike and foot-off) and walking speed during each stride and averaged the results. Step symmetry was expressed as the ratio of the larger stepping length value in the numerator per recommendation (Patterson et al., 2010). This asymmetry reflects a compensatory reliance on using the less-affected side to generate body propulsion forces (Roelker et al., 2019).

### Sample size

Expecting a dropout rate of 25%, we aimed to enroll 40 participants to achieve 15 participants per study arm, as recommended for pilot studies (Whitehead et al., 2016). We scheduled 20 months of recruitment, expecting to recruit two participants/month.

### Statistics

Demographic, disease-specific, and intervention descriptors are reported as medians (range Q1–Q3) in case of continuous variables, or as amounts (*n*) for ordinal and nominal variables. For our analyses, FM-LE, COPvel-ap, COPvel-ml, WBA, DCA, stepping symmetry, and walking speed were treated as continuous variables; BBS-stand and FAC were treated as ordinal scales and descriptively analyzed only.

To test whether FM-LE recovery courses were different between groups, linear mixed models were applied, including fixed effects for GROUP (ERT + UC, DRT + UC), TIME (baseline, week 5, week 8, week 12), and GROUPxTIME interaction, and a subject-specific random intercept. This yielded β-coefficients with their standard error and confidence interval (CI) reflecting a TIME effect *within* groups and a GROUPxTIME interaction showing differences in FM-LE change *between* groups during the early period ranging from baseline to week 5 poststroke (i.e., ERT with UC vs. UC), from weeks 5 to 8 poststroke (i.e., sustainability of ERT), and during the delayed period ranging from weeks 8 to 12 poststroke (i.e., DRT with UC vs. UC). The model was tested for normality assumptions using Q-Q plots. Homoskedasticity was checked using a plot of residuals by predicted values.

To deal with the absence of biomechanical data in nonambulatory participants during the first assessment, thereby limiting time course analyses, we analyzed between*-*group differences in COPvel-ap, COPvel-ml, WBA, DCA, stepping symmetry, and walking speed in a different way. Here, we calculated the mean difference with CI using independent sample Welch's *t*-tests (assuming unequal variance) cross-sectionally at each time point. T-tests were applied if data from at least 50% of the sample were available.

By lack of established thresholds of clinically meaningful differences, we decided that a 15% difference in outcomes would be required to confirm our hypotheses. It was understood that the trial would not be adequately powered to detect this with statistical significance. Therefore, to protect against premature rejection of a potential benefit, we relied on descriptives and trends by CIs of varying widths per recommendation (Lee et al., 2014). Thus, we not only used the two-tailed traditional α-rate (Type I error, false-positive) of 0.05 but also included results based on a α-rate of 0.15. This resulted in an 85%CI, besides the traditional 95%CI.

All statistical analyses were performed using JMP Pro v16 software (SAS, NC, USA). Because this was a pilot study, the preliminary analyses were restricted to participants who completed the intervention (i.e., on-treatment analyses).

## Results

### Recruitment

**Figure 2:**
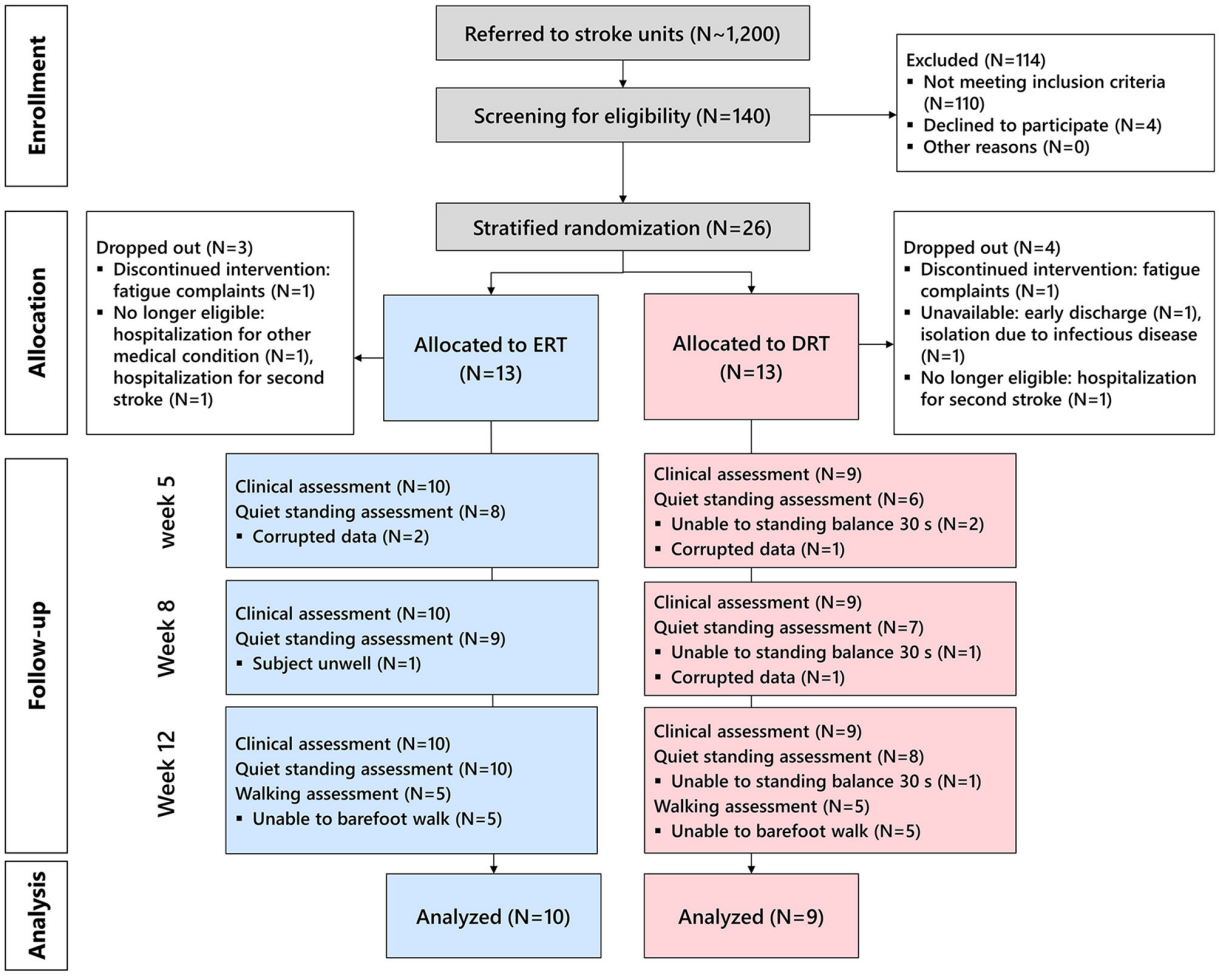
CONSORT flow diagram of the inclusion and follow-up. ERT, early robotic training; DRT, delayed robotic training. After screening, 140 potential candidates were identified, of which 26 were enrolled, stratified, and randomly allocated. Eventually, 19 participants successfully underwent the intervention and follow-up measurements and were included in the on-treatment analysis.

Figure 2 shows the inclusion flow. Between December 2018 and November 2021, ~1,200 patients were screened upon hospital admission. Screening was interrupted from February 2020 to September 2021 because of restricted hospital access due to COVID-19 measures. During the 15 months of actively recruiting, 140 potential candidates were identified, and 26 participants were enrolled (1.73 participants/month). The main exclusion reasons were “too mild impairments” (i.e., NIHSS motor leg item < 1 and/or FAC >3) and “short length of stay” resulting in immediate discharge. The trial was eventually stopped due to expiration of funding.

Of the 26 enrolled patients, 19 successfully participated (dropout rate 28%) in the ERT (*N* = 10) or DRT (*N* = 9) groups. Seven dropouts were registered: two subjects had a second stroke, one subject suffered another sudden-onset disease limiting participation, one subject was unavailable after discharge, one subject was isolated due to an infectious disease, and two subjects discontinued robotic training.

The 19 included participants had a median age of 64 (52–76) years and body weight of 70 (46–84) kg. Nine were female (47%), 16 had an ischemic stroke (84%), and 13 suffered left-sided weakness (68%). Median baseline FM-LE and MI-LE scores were 14 (6.5–21.5) and 37 (27–47), respectively. At baseline, 13 participants had sitting balance (68%) and one participant (5%) could stand independently. According to the EPOS model (see above), 11 participants had a favorable prognosis for walking (58%), and eight had a poor prognosis (42%). The ERT and DRT arms were comparable in these baseline variables (Table 1).

**Table 1:** Baseline characteristics of participants and intervention characteristics.

	**ERT**	**DRT**	***P*-value**
	***N*** = **10**	***N*** = **9**	
**Demographics and stroke information**			
Age (years)	60.0 (53.0–82.3)	69.0 (48.5–76.0)	0.987
Sex (female/male)^*^	4/6	5/4	0.66
Body weight (kg)	66.9 (48.4–80.2)	70.7 (63.15–80.15)	0.35
Paretic body side (left/right)^*^	6/4	7/2	0.63
Stroke type (ischemic/hemorrhage)^*^	9/1	7/2	0.58
**Care characteristics**			
Length of stay in inpatient rehabilitation (weeks)	15.0 (14.0–23.5)	19.0 (15.5–25.5)	0.62
Discharge destination after rehabilitation (home/nursing facility or other hospital)^*^	6/4	8/1	0.29
**Clinical characteristics**			
FM-LE (0–34)	15.0 (8.5–20.5)	11.0 (6.0–19.5)	0.56
MI-LE (0–99)	42.0 (23.0–53.0)	33.0 (23.0–55.5)	0.74
FAC 0 (*n*)^*^	9	7	NA
FAC 1 (*n*)^*^	1	2	NA
TCT-sit: able to sit for 30 s? (yes/no)^*^	6/4	7/2	0.63
BBS-stand: able to stand 30 s? (yes/no)^*^	1/9	0/9	1
Prognosis of walking ability (favorable/poor)^*^	6/4	5/4	1
**Intervention characteristics**			
Number of training sessions (*n*)	16 (15–16)	16 (16–16)	0.21
Time spent practicing posture and gait per session (min)	22.7 (19.8–25.5)	20.8 (24.4–18.5)	0.37
Practice steps taken per session (*n*)	856 (735–918)	866 (697–908)	0.97

ERT, early robotic training study arm; DRT, delayed robotic training study arm; FM-LE, Fugl-Meyer Lower Extremity motor scores; MI-LE, Motricity Index Lower Extremity score; FAC, Functional Ambulation Category; TCT-sit, Trunk Control Test—sitting item; BBS-stand, Berg Balance Scale—unsupported standing item, NA, not applicable as data is only displayed for inspection.

Values are presented as medians (range Q1–Q3) in case of continuous variables or counts in case of nominal variables (marked with ^*^). Acknowledging the limited sample size, non-parametric tests were used to compare the groups and *P*-values were derived from Wilcoxon rank sum tests or Fisher's exact tests (^*^).

Measurements were applied at baseline, which ranged from 7 to 14 days poststroke; at week 5 after completion of ERT, which ranged from 36 to 44 days poststroke; at week 8, ranging from 57 to 63 days poststroke; and at week 12 after completion of DRT, which ranged from 84 to 94 days poststroke. The timings were identical between groups (*P* > 0.05).

### Intervention

Two participants, one in each group, felt too exhausted to train above UC. Among the subjects who completed robotic training, 17 received all 16 sessions, and two received 14 sessions due to scheduling issues. Over the entire 300 robotic sessions, 10 cases (3%) of negative side effects were documented (ERT: *N* = 6 vs. DRT: *N* = 4) including minor joint pain and sore muscles, leading to a temporal training intensity reduction. No adverse events were recorded.

The median practice time per session in ERT vs. DRT was 22.7 (19.8–25.6) vs. 20.8 (17.8–23.8) min, and the steps/session were 856 (765–948) vs. 866 (761–971).

### Preliminary treatment effects on clinical outcomes

**Figure 3:**
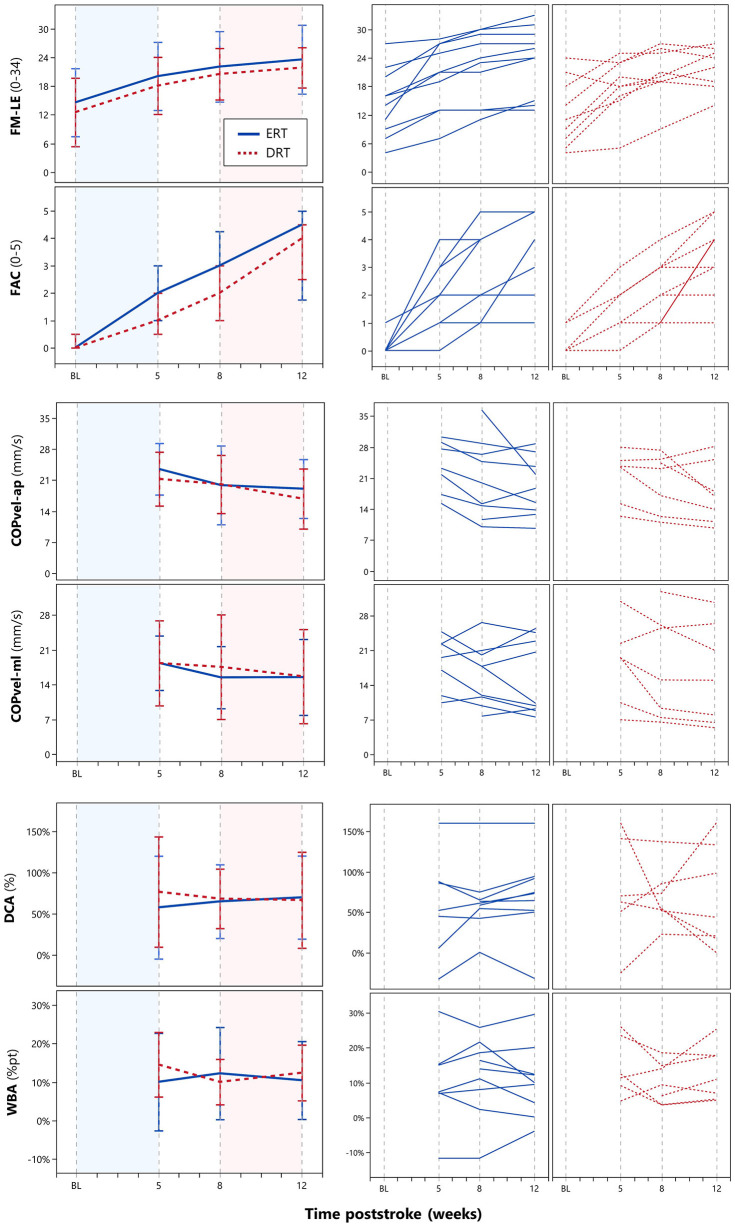
Time course of intralimb muscle synergies (FM-LE), walking independence (FAC), and postural stability (COPvel-ap, COPvel-ml) and interlimb asymmetries (DCA, WBA) during quiet bipedal standing. FM-LE, Fugl-Meyer Lower Extremity motor scores; FAC, functional ambulation category; ERT, early robotic training; DRT, delayed robotic training. The left line graphs display means and their standard deviations as error bars for continuous variables (FM-LE, COPvel-ap, COPvel-ml, DCA, WBA) and median with interquartile range for a nominal variable (FAC) for both ERT participants (*N* = 10) represented by blue solid lines and DRT participants (*N* = 9) represented by red dotted lines. The x-axes represent the time poststroke in weeks with fixed time points of assessment at baseline (BL, <14 days poststroke) and weeks 5, 8, and 12 poststroke follow-up. The epoch marked in blue, spanning from BL to week 5, represents the early intervention period (ERT with UC vs. UC). The epoch marked in red, spanning from week 8 to week 12, represents the delayed intervention period (DRT with UC vs. UC). The graphs on the right are similarly designed, but they display individual recovery curves.

A figure with the mean and individual time courses is provided as Figure 3. Inspection yields parallel recovery courses between groups, yet a tendency toward faster FAC gains during the early period favoring ERT with UC over UC.

Table 2 summarizes TIME and GROUPxTIME effects on FM-LE change. As shown, there was a significant TIME effect during the early period *within* the ERT and DRT arms at α = 0.05 [β = 5.50, 95% CI (3.12; 7.88) and β = 5.56, 95% CI (3.05; 8.06)]. Change leveled off thereafter. The GROUPxTIME interaction effect was not significant.

**Table 2:** Effects of the TIME and GROUP × TIME interactions on the recovery of intralimb muscle synergies in the most-affected lower limb.

		**Early period: baseline to week 5**			**Week 5 to week 8**			**Delayed period: week 8 to week 12**		
		**TIME (ERT)**	**TIME (DRT)**	**GROUP** × **TIME (difference)**	**TIME (ERT)**	**TIME (DRT)**	**GROUP** × **TIME (difference)**	**TIME (ERT)**	**TIME (DRT)**	**GROUP** × **TIME (difference)**
FM-LE (0–34)	β (SE)	**5.50** ^*^ **(1.86)**	**5.56** ^*^ **(1.25)**	−0.06 (1.72)	**2.00** ^**×**^ **(1.19)**	**2.44** ^**×**^ **(1.25)**	−0.44 (1.72)	1.50 (1.86)	1.33 (1.25)	0.17 (1.72)
	95% CI	**3.12; 7.88**	**3.05; 8.06**	−3.51; 3.40	−0.38; 4.38	−0.06; 4.95	−3.90; 3.01	−0.88; 3.88	−1.18; 3.84	−3.29; 3.63
	85% CI	**3.77; 7.23**	**3.73; 7.38**	−2.57; 2.46	**0.27; 3.73**	**0.62; 4.27**	−2.96; 2.07	−0.23; 3.23	−0.49; 3.16	−2.35; 2.68

ERT, early robotic training study arm; DRT, delayed robotic training study arm; FM-LE, Fugl-Meyer Lower Extremity motor scores.

Estimated regression coefficients (β), standard error (SE), and confidence interval (CI) at certainty levels of 95% and 85% are shown. TIME effects represent FM-LE change within ERT (*N* = 10) and DRT (*N* = 9) study arms. A GROUP × TIME interaction represents between group differences in FM-LE change. Marked β-coefficients reflect a statistically significant effect with ^*^α-rate = 0.05 and ^×^α-rate = 0.15. CIs in **bold** do not include zero, the value expected under the null hypothesis.

The distribution of BBS-stand FAC scores is illustrated in Table 3. As shown, the number of subjects achieving FAC ≥4 in the ERT vs. DRT arms was 1 (10%) vs. 0 (0%) at week 5; 5 (50%) vs. 1 (11%) at week 8; and 6 (60%) vs. 5 (56%) at week 12.

**Table 3:** Mean scores and between-group differences in the clinical and biomechanical outcomes of quiet standing balance and walking performance.

	**Baseline**		**Week 5**			**Week 8**			**Week 12**		
	**ERT**	**DRT**	**ERT**	**DRT**	**Difference (95%CI) (85%CI)**	**ERT**	**DRT**	**Difference (95%CI) (85%CI)**	**ERT**	**DRT**	**Difference (95%CI) (85%CI)**
**Task: quiet standing**											
BBS-stand 0 (*n*)	9	8	0	1		0	0		0	0	
BBS-stand 1 (*n*)	0	1	1	1		0	0		0	0	
BBS-stand 2 (*n*)	1	0	1	0		2	2		1	1	
BBS-stand 3 (*n*)	0	0	2	3		2	2		3	0	
BBS-stand 4 (*n*)	0	0	6	4		6	5		6	8	
BBS-stand ≥ 2 (*n*)	1	0	9	7		10	9		10	9	
Instrumented measurement available (*n*)	1	0	8	6		9	7		10	8	
COPvel-ap (mm/s^2^)			23.50 (5.81)	21.28 (6.08)	2.27 (−5.11; 9.56) (−2.92; 7.37)	19.87 (8.87)	20.08 (6.56)	−0.21 (−8.86; 8.45) (−6.33; 5.92)	19.07 (6.64)	16.81 (6.76)	2.26 (−4.69; 9.21) (−2.69; 7.21)
COPvel-ml (mm/s^2^)			18.39 (5.48)	18.37 (8.56)	0.02 (−9.29; 9.34) (−6.42; 6.47)	15.45 (6.26)	17.57 (10.53)	−2.12 (−12.33; 8.11) (−9.25; 5.02)	15.51 (7.64)	15.67 (9.48)	−0.16 (−9.22; 8.90) (−6.58; 6.27)
DCA (%)			57.75 (62.19)	76.60 (66.68)	−18.84 (−98.56; 60.87) (−74.74; 37.06)	64.96 (44.60)	68.28 (35.99)	−3.32 (−48.33; 41.70) (−35.19; 28.55)	69.84 (50.27)	66.63 (58.06)	3.20 (−53.63; 60.04) (−37.16; 43.57)
WBA (%pt)			10.03 (12.63)	14.52 (8.37)	−4.49 (−17.50; 8.53) (−13.62; 4.64)	12.24 (11.94)	10.02 (5.89)	2.21 (−8.35; 12.78) (−5.20; 9.26)	10.45 (10.08)	12.39 (7.20)	−1.95 (−19.96; 7.07) (−8.36; 4.46)
**Task: walking**											
FAC 0 (*n*)	9	7	1	2		0	0		0	0	
FAC 1 (*n*)	1	2	3	3		3	3		2	1	
FAC 2 (*n*)	0	0	2	3		2	2		1	1	
FAC 3 (*n*)	0	0	3	1		0	3		1	2	
FAC 4 (*n*)	0	0	1	0		3	1		1	3	
FAC 5 (*n*)	0	0	0	0		2	0		5	2	
FAC ≥4 (*n*)	0	0	1	0		5	1		6	5	
Instrumented measurement available (*n*)	0	0	1	1		4	3		5	5	
Walking speed (m/s)									0.74 (0.32)	0.56 (0.26)	0.17 (−0.25; 0.60) (−0.12; 0.47)
Stepping symmetry (ratio)									1.065 (0.052)	1.050 (0.053)	0.015 (−0.062; 0.092) (−0.038; 0.069)

ERT, early robotic training study arm; DRT, delayed robotic training study arm; SD, standard deviation; CI, confidence interval; BBS-stand, standing unsupported item of the Berg Balance Scale; COPvel-ap, net COP sway velocity in the anteroposterior direction; COPvel-ml, net COP sway velocity in the mediolateral direction; DCA, dynamic control asymmetry; WBA, weight-bearing asymmetry; FAC, functional ambulation category; SD, standard deviation.

BBS-stand and FAC are ordinal scales, and the distribution of scores is shown as counts (n) for inspection. The number of available biomechanical measurements is shown similarly as counts (n). Subsequently, biomechanical outcomes are represented as means (SD) for the ERT (*N* = 10) and DRT (*N* = 9) groups separately, and mean differences between groups with CIs at certainty levels of both 95% (α-rate = 0.05) and 85% (α-rate = 0.15).

### Preliminary treatment effects on biomechanical outcomes

There were missing data from quiet standing measurements at week 5, as two subjects had poor balance, and three had to be excluded because of corrupted datasets. At week 8, one subject declined to participate, and one measurement was lost due to a software error. A single participant could not perform the measurements at weeks 8 and 12 (Figure 2). Hence, eligible balance data in ERT vs. DRT were *N* = 8 (80%) vs. *N* = 6 (67%) at week 5; *N* = 9 (90%) vs. *N* = 7 (78%) at week 8; and *N* = 10 (100%) vs. *N* = 8 (89%) at week 12. As summarized in Table 3, the mean differences in COPvel-ap, COPvel-ml, DCA, and WBA were non-significant at weeks 5, 8, and 12 using either α-rate.

At week 12, 14 participants reached FAC ≥3 whereas four required support to barefoot walk following our protocol. Therefore, 10 participants (53%), five in each group, could be tested at week 12, allowing for a between-group comparison. This resulted in non-significant mean differences in stepping symmetry and walking speed using either α-rate (Table 3).

## Discussion

This pilot study showed that patients with severe walking limitations (i.e., FAC < 2) at baseline tolerated overground training with the Ekso GT^®^ in addition to UC for 4 weeks without any adverse events reported. Only two of 21 participants (9.5%) who received such robotic training withdrew because of exhaustion-related complaints.

Comparing both groups, we found a trend toward earlier independent walking, consistent with the general lower limb robotics literature (Schroder et al., 2019; Mehrholz et al., 2020). Five patients (50%) who underwent ERT with UC within the first 5 weeks poststroke achieved independence, or FAC ≥4, by week 8 whereas only one participant in the control group (11%) did. However, regarding our first objective, this potential benefit was not accompanied by greater improvements in FM-LE, and both groups exhibited comparable postural stability (COPvel-ap, COPvel-ml) and a strategy to predominantly maintain balance on the less-affected side (WBA, DCA). Thereafter, DRT with UC shows similarly to be invariant in terms of improving FM-LE scores or any standing balance metric relative to UC alone. During the final assessment, both groups also exhibited a very low self-selected walking speed below the cut-offs of 0.8–0.9 m/s, which are indicative of community-based walking after stroke (Bowden et al., 2008; Fulk et al., 2017). This suggests that the unexpected stepping symmetry reflects a “cautious gait” (Giladi et al., 2005; Herman et al., 2005) rather than the re-emergence of a normal, efficient walking pattern.

To date, the question of *when* to intervene with stroke rehabilitation therapies has only been specifically addressed by animal models, which suggest a time-sensitive period for intensive motor training in the early poststroke period (Krakauer et al., 2012; Zeiler, 2019). Recently, the CPASS trial has provided some clinical evidence that delivering additional 20 h of arm-hand skill training during the acute and early subacute phases can boost recovery on the Action Research Arm Test, compared with additional training starting >6 months poststroke (Dromerick et al., 2015, 2021). However, the trial lacked measurements adequately reflecting impairment reductions, such as Fugl-Meyer scores or performance assays. This omission is important to address because the applied scale does not provide insight into whether therapy-induced improvements were achieved by behavioral restitution or compensation (Kwakkel et al., 2019; Savitz, 2022). Therefore, to capture interaction effects for restoring quality of movement, we have consciously applied instrumented biomechanical measurements of quiet standing balance and walking, including estimates of interlimb symmetry. However, we must acknowledge that currently validated performance assays reflecting behavioral restitution of the lower limb are lacking, as was established at the third SRRR (Van Criekinge et al., 2024). In this context, we assumed that muscle synergies, as measured with FM-LE, closely associate with “true” motor recovery by reflecting intra-limb coordination and selectively controlling the lower limb voluntarily (Van Criekinge et al., 2024). However, we did not identify any trends supporting our hypothesis that muscle synergies can be corrected through 4 weeks of additional training with an exoskeleton to reduce asymmetries and improve performance of quiet standing and walking tasks. Additionally, the inability to restore synergy-independent movement control appears to be independent of the timing of robotic training, whether it is within (i.e., ERT) or after (i.e., DRT) the critical period of most spontaneous motor recovery.

### Perspectives for future robotic rehabilitation research

To our knowledge, four other small-scaled randomized trials investigated the effects of incorporating novel wearable exoskeletons for overground stepping practice (Ekso GT^®^ or HAL^®^) in primary inpatient rehabilitation < 3 months poststroke, either additional to (Molteni et al., 2021) or embedded in (Wall et al., 2020; Louie et al., 2021; Yokota et al., 2023) UC. Similar to our study, they included nonambulatory patients due to a trend toward greater benefit in this subgroup (Mehrholz et al., 2020). Nevertheless, these trials were not successful in showing superiority over equally-dose conventional therapies on the primary outcomes, being the 6-min walk test (Molteni et al., 2021) and FAC (Wall et al., 2020; Louie et al., 2021; Yokota et al., 2023). Additionally, non-randomized observations in small samples of inpatients who underwent overground exoskeleton training did not show trends toward normalization of EMG muscle coordination patterns (Infarinato et al., 2021) or kinematic gait profile scores (Wall et al., 2023), as other performance assays thought to reflect quality of lower limb movement poststroke (Van Criekinge et al., 2024).

The key question is *what* and *how* do patients learn to accomplish clinically meaningful tasks during training with exoskeletons. One concern with current robots, whether stationary (i.e., footplates- or treadmill-based) or mobile (i.e., overground), is that they impose a predetermined stepping trajectory, which limits movement variability and the ability to learn from mistakes as “error signals.” Consequently, patients must adapt to the exoskeleton, hindering the acquisition of necessary compensation strategies. Additionally most robots, such as the Ekso GT^®^, focus on hip and knee kinematics, potentially disregarding the training of ankle movements that are integral for steady-state balance control (Roelofs et al., 2018) and achieving higher walking speeds (Roelker et al., 2019). The apparent need for compensation to regain safety and independence in activities such as bipedal standing (Laufer et al., 2003; Garland et al., 2007; Roerdink et al., 2009; Schroder et al., 2023) and walking (Kwakkel and Wagenaar, 2002; Buurke et al., 2008; Patterson et al., 2015), raises the key question of whether robots should aim to correct kinematics. Therefore, to gain more insight into *what* and *how* patients learn during rehabilitation, future trials should include serially applied kinetics and/or kinematics. Our preliminary findings show that despite improvements in postural stability and FAC, asymmetries persisted (see Figure 3). This is consistent with aforementioned longitudinal studies (Kwakkel and Wagenaar, 2002; Laufer et al., 2003; Garland et al., 2007; Buurke et al., 2008; Roerdink et al., 2009; Patterson et al., 2015; Schroder et al., 2023), as functional improvements seem to be mainly a matter of behavioral compensation, where patients learn to adapt to their existing deficits. This suggests the need for a new generation of ‘assist-as-needed' robotic designs with greater degrees of freedom, enabling patients to gradually learn compensations. Ideally, devices should be applicable in ecologically valid environments to facilitate the transfer of trained skills from the robotic milieu to real-world performance in a patient's home and nearby community.

### Limitations

The main limitation was the underpowered, small sample that was unbalanced in FM-LE baseline scores, slightly favoring ERT. We did not reach the desired sample of 15 participants/group as recruitment was hindered during the COVID-19 pandemic and was slower than expected (1.73 vs. 2 subjects/month). However, this agrees with a recruitment analysis of CPASS (Geed et al., 2021) showing a low rate of 1–2 participants/month in an acute hospital setting also applying perfusion therapies. Other limitations include our definition of the sensitive period, which is somewhat arbitrary, not knowing the exact moment to intervene at a patient level. Additionally, we were largely restricted to a standing task for assessing symmetry. Therefore, it is important to validate protocols for collecting gait biomechanics more broadly, e.g., by allowing footwear and orthotics (Patterson et al., 2015) or even light support (Henderson et al., 2022), ideally with portable technology. Our findings should be considered in the context of a specific intervention protocol, which may not generalize to other devices. Likewise, although we were able to deliver 4 weeks of training with 800–900 steps/session, thereby exceeding step counts during conventional therapies (Lang et al., 2009; Rand and Eng, 2012; Kuys et al., 2019), it is unclear whether our results would have differed had the training dose been even increased and prolonged. In that regard, it is important to mention that recent randomized trials illustrate that dosing >2,000 steps/session may be required for improving walking capacity poststroke (Hornby et al., 2016; Klassen et al., 2020; Boyne et al., 2023). We finally acknowledge the limitation of not documenting UC and daily walking performance beyond the protocolized intervention. After all, robotic training could have competed with UC for patients' time and energy, potentially resulting in a leveling-off of the intended treatment contrast in terms of augmented exercise time. To overcome this limitation, future trials may benefit from using daily step count and activity trackers to determine the actual amount of exercise each participant performed.

## Conclusion

This pilot study tested the feasibility and preliminary effects of a 4-week overground training program using the Ekso GT^®^ wearable exoskeleton as an adjunct to UC in nonambulatory patients, applied either early (i.e., within 5 weeks) or delayed (i.e., at 8 weeks) after stroke onset. The intervention had a high adherence rate; however, we identified barriers to patient recruitment and biomechanical data collection that may limit a larger phase II trial. Outcomes collected in this small sample do not support such a resource-intensive trial, as we did not find any trend in between-group differences that favored lower limb motor recovery (FM-LE) with additional robotic training to promote symmetry and improve quiet standing and walking performance. Furthermore, our findings suggest that the effectiveness of our intervention was not modulated by an early or delayed timing. With that, the surplus value of therapeutic robots that attempt to restore “normal” movement for stroke rehabilitation has been further questioned (Hornby, 2022; Dobkin and Busza, 2023; Putrino and Krakauer, 2023).

## Data availability statement

The raw data supporting the conclusions of this article are available as a supplementary file (see Supplementary material). Further requests to the corresponding author will be considered without undue reservation.

## Ethics statement

The studies involving humans were approved by Ethics Committee UZA/UAntwerpen. The studies were conducted in accordance with the local legislation and institutional requirements. The participants provided their written informed consent to participate in this study.

## Author contributions

JS: Conceptualization, Data curation, Formal analysis, Funding acquisition, Project administration, Writing – original draft, Writing – review & editing. LY: Conceptualization, Supervision, Writing – review & editing. EE: Data curation, Writing – review & editing. RL-C: Data curation, Writing – review & editing. AH: Methodology, Software, Writing – review & editing. CL: Resources, Writing – review & editing. ST: Conceptualization, Funding acquisition, Supervision, Writing – review & editing. GK: Conceptualization, Supervision, Writing – original draft, Writing – review & editing. WS: Conceptualization, Funding acquisition, Project administration, Supervision, Writing – review & editing, Writing – original draft.

## Footnotes

^1^ https://strokerecoveryalliance.com/
