# Supplementary material for: Exoskeleton-assisted training to enhance lower limb motor recovery in subacute stroke: does timing matter? A pilot randomized trial
Source: Front Stroke. 2024 May 14;3:1379083. doi: 10.3389/fstro.2024.1379083 (PMC12802626; doi:10.3389/fstro.2024.1379083)
Supplement: Supplementary file 1 [file Data_Sheet_1.PDF]

ID	Age (years)	Sex	Weight (kg)	Stroke type	Body side affected	Length of rehabilitation stay (weeks)	Discharge destination	EPOS prognosis	Allocation	EXO sessions (n)	EXO start (days poststroke)	EXO duration (days)	EXO steps/session	EXO upright time/session (min)	EXO walking time/session (min)	Assessment session	CLIN timing (days poststroke)	FM-LE	MI-LE	MI_hip	MI_knee
4	57	F	58.4	i	L	19	home	Fav	DRT	16	63	27	906.125	24.375	22.4375	baseline	8	14	37	14	14
4	57	F	58.4	i	L	19	home	Fav	DRT	16	63	27	906.125	24.375	22.4375	5w	40	23	37	14	14
4	57	F	58.4	i	L	19	home	Fav	DRT	16	63	27	906.125	24.375	22.4375	8w	62	26	42	14	14
4	57	F	58.4	i	L	19	home	Fav	DRT	16	63	27	906.125	24.375	22.4375	12w	92	24	58	19	25
5	61	M	49.2	i	L	14	home	Fav	ERT	15	16	25	926	24.4	21.4	baseline	12	11	42	14	14
5	61	M	49.2	i	L	14	home	Fav	ERT	15	16	25	926	24.4	21.4	5w	40	27	75	25	25
5	61	M	49.2	i	L	14	home	Fav	ERT	15	16	25	926	24.4	21.4	8w	60	29	91	33	33
5	61	M	49.2	i	L	14	home	Fav	ERT	15	16	25	926	24.4	21.4	12w	88	29	91	33	33
6	56	F	47	h	R	15	home	Fav	ERT	15	15	24	856.7333333	21.93333333	19.06666667	baseline	11	22	42	14	14
6	56	F	47	h	R	15	home	Fav	ERT	15	15	24	856.7333333	21.93333333	19.06666667	5w	38	25	75	25	25
6	56	F	47	h	R	15	home	Fav	ERT	15	15	24	856.7333333	21.93333333	19.06666667	8w	59	27	69	25	25
6	56	F	47	h	R	15	home	Fav	ERT	15	15	24	856.7333333	21.93333333	19.06666667	12w	91	27	75	25	25
7	50	M	87.4	i	R	26	care facility	Poor	ERT	13	18	25	915.9230769	33.30769231	26.53846154	baseline	14	16	37	14	14
7	50	M	87.4	i	R	26	care facility	Poor	ERT	13	18	25	915.9230769	33.30769231	26.53846154	5w	40	19	53	14	25
7	50	M	87.4	i	R	26	care facility	Poor	ERT	13	18	25	915.9230769	33.30769231	26.53846154	8w	63	23	57	19	19
7	50	M	87.4	i	R	26	care facility	Poor	ERT	13	18	25	915.9230769	33.30769231	26.53846154	12w	90	24	69	19	25
8	24	F	78	i	R	17	home	Poor	DRT	16	56	29	789.25	20	17.125	baseline	14	5	23	9	14
8	24	F	78	i	R	17	home	Poor	DRT	16	56	29	789.25	20	17.125	5w	38	16	33	19	14
8	24	F	78	i	R	17	home	Poor	DRT	16	56	29	789.25	20	17.125	8w	62	19	59	25	25
8	24	F	78	i	R	17	home	Poor	DRT	16	56	29	789.25	20	17.125	12w	92	18	64	25	25
9	71	M	70	h	R	29	home	Poor	DRT	16	58	24	989.5	24.875	22.25	baseline	10	4	0	0	0
9	71	M	70	h	R	29	home	Poor	DRT	16	58	24	989.5	24.875	22.25	5w	39	5	9	9	0
9	71	M	70	h	R	29	home	Poor	DRT	16	58	24	989.5	24.875	22.25	8w	59	9	37	14	14
9	71	M	70	h	R	29	home	Poor	DRT	16	58	24	989.5	24.875	22.25	12w	88	14	37	14	14
10	59	M	80.9	i	L	21	rehab facility	Fav	ERT	16	18	27	862.3125	26.4375	22	baseline	15	16	42	14	14
10	59	M	80.9	i	L	21	rehab facility	Fav	ERT	16	18	27	862.3125	26.4375	22	5w	38	21	47	19	14
10	59	M	80.9	i	L	21	rehab facility	Fav	ERT	16	18	27	862.3125	26.4375	22	8w	58	24	75	25	25
10	59	M	80.9	i	L	21	rehab facility	Fav	ERT	16	18	27	862.3125	26.4375	22	12w	86	26	75	25	25
11	54	M	69.9	i	R	46	home	Poor	ERT	16	18	24	856.125	25.1875	21.125	baseline	14	4	0	0	0
11	54	M	69.9	i	R	46	home	Poor	ERT	16	18	24	856.125	25.1875	21.125	5w	41	7	28	14	14
11	54	M	69.9	i	R	46	home	Poor	ERT	16	18	24	856.125	25.1875	21.125	8w	63	11	42	14	14
11	54	M	69.9	i	R	46	home	Poor	ERT	16	18	24	856.125	25.1875	21.125	12w	88	15	42	14	14
17	81	M	63.9	i	L	21	care facility	Fav	ERT	16	12	25	943.6875	23.5625	18.5625	baseline	8	27	75	25	25
17	81	M	63.9	i	L	21	care facility	Fav	ERT	16	12	25	943.6875	23.5625	18.5625	5w	36	28	83	25	25
17	81	M	63.9	i	L	21	care facility	Fav	ERT	16	12	25	943.6875	23.5625	18.5625	8w	58	30	83	25	33
17	81	M	63.9	i	L	21	care facility	Fav	ERT	16	12	25	943.6875	23.5625	18.5625	12w	85	31	75	25	25
20	74	F	52.1	i	L	21	home	Fav	DRT	16	56	25	909	21.625	19.6875	baseline	14	11	32	9	14
20	74	F	52.1	i	L	21	home	Fav	DRT	16	56	25	909	21.625	19.6875	5w	42	15	47	14	14
20	74	F	52.1	i	L	21	home	Fav	DRT	16	56	25	909	21.625	19.6875	8w	61	21	42	14	14
20	74	F	52.1	i	L	21	home	Fav	DRT	16	56	25	909	21.625	19.6875	12w	88	19	69	19	25
21	86	F	46.4	i	L	14	care facility	Poor	ERT	16	13	30	735.625	19.375	15.4375	baseline	9	7	23	9	14
21	86	F	46.4	i	L	14	care facility	Poor	ERT	16	13	30	735.625	19.375	15.4375	5w	41	13	42	14	19
21	86	F	46.4	i	L	14	care facility	Poor	ERT	16	13	30	735.625	19.375	15.4375	8w	62	13	28	14	14
21	86	F	46.4	i	L	14	care facility	Poor	ERT	16	13	30	735.625	19.375	15.4375	12w	94	14	37	14	14
24	40	M	70.7	h	L	17	home	Fav	DRT	16	56	26	902	24.4375	20.5625	baseline	13	24	75	25	25
24	40	M	70.7	h	L	17	home	Fav	DRT	16	56	26	902	24.4375	20.5625	5w	40	23	75	25	25
24	40	M	70.7	h	L	17	home	Fav	DRT	16	56	26	902	24.4375	20.5625	8w	57	27	75	25	25
24	40	M	70.7	h	L	17	home	Fav	DRT	16	56	26	902	24.4375	20.5625	12w	84	26	75	25	25
28	64	M	88.4	i	L	14	home	Poor	DRT	16	65	29	752	19	17.3125	baseline	14	7	23	9	14
28	64	M	88.4	i	L	14	home	Poor	DRT	16	65	29	752	19	17.3125	5w	38	18	42	14	14
28	64	M	88.4	i	L	14	home	Poor	DRT	16	65	29	752	19	17.3125	8w	57	20	58	19	25
28	64	M	88.4	i	L	14	home	Poor	DRT	16	65	29	752	19	17.3125	12w	93	25	72	25	33
29	69	M	67.9	i	L	11	home	Fav	DRT	16	58	24	866.3125	20.8125	19.0625	baseline	11	21	53	14	25
29	69	M	67.9	i	L	11	home	Fav	DRT	16	58	24	866.3125	20.8125	19.0625	5w	44	18	42	14	19
29	69	M	67.9	i	L	11	home	Fav	DRT	16	58	24	866.3125	20.8125	19.0625	8w	60	19	53	25	14
29	69	M	67.9	i	L	11	home	Fav	DRT	16	58	24	866.3125	20.8125	19.0625	12w	85	22	64	25	25
30	89	M	73.8	i	L	11	home	Fav	ERT	16	11	28	734.75	20	17.5	baseline	7	14	53	25	14
30	89	M	73.8	i	L	11	home	Fav	ERT	16	11	28	734.75	20	17.5	5w	36	21	42	14	14
30	89	M	73.8	i	L	11	home	Fav	ERT	16	11	28	734.75	20	17.5	8w	58	21	75	25	25
30	89	M	73.8	i	L	11	home	Fav	ERT	16	11	28	734.75	20	17.5	12w	91	24	99	33	33
34	78	F	82.3	i	L	28	care facility	Poor	DRT	16	63	26	622.6875	15.875	14	baseline	12	18	58	14	25
34	78	F	82.3	i	L	28	care facility	Poor	DRT	16	63	26	622.6875	15.875	14	5w	41	25	64	25	25
34	78	F	82.3	i	L	28	care facility	Poor	DRT	16	63	26	622.6875	15.875	14	8w	60	25	83	25	33
34	78	F	82.3	i	L	28	care facility	Poor	DRT	16	63	26	622.6875	15.875	14	12w	89	27	91	33	33
37	68	F	48.9	i	R	15	home	Fav	ERT	16	15	27	804.875	20.5	18	baseline	13	20	53	14	14
37	68	F	48.9	i	R	15	home	Fav	ERT	16	15	27	804.875	20.5	18	5w	38	27	91	25	33
37	68	F	48.9	i	R	15	home	Fav	ERT	16	15	27	804.875	20.5	18	8w	58	30	99	33	33
37	68	F	48.9	i	R	15	home	Fav	ERT	16	15	27	804.875	20.5	18	12w	87	33	91	25	33
39	82	F	70.7	i	L	23	home	Fav	DRT	16	58	30	642.875	17.9375	11.25	baseline	9	9	33	14	19
39	82	F	70.7	i	L	23	home	Fav	DRT	16	58	30	642.875	17.9375	11.25	5w	39	20	47	14	19
39	82	F	70.7	i	L	23	home	Fav	DRT	16	58	30	642.875	17.9375	11.25	8w	60	19	42	14	14
39	82	F	70.7	i	L	23	home	Fav	DRT	16	58	30	642.875	17.9375	11.25	12w	89	22	52	14	19
94	45	F	80	i	L	n/r	n/r	Poor	ERT	16	17	35	224.1875	10.0625	6.6875	baseline	13	9	23	9	14
94	45	F	80	i	L	n/r	n/r	Poor	ERT	16	17	35	224.1875	10.0625	6.6875	5w	42	13	32	9	14
94	45	F	80	i	L	n/r	n/r	Poor	ERT	16	17	35	224.1875	10.0625	6.6875	8w	59	13	28	14	14
94	45	F	80	i	L	n/r	n/r	Poor	ERT	16	17	35	224.1875	10.0625	6.6875	12w	89	13	33	14	19

MI_ankle	TCT-s	BBS-s	FAC	BALANS assessment available	BALANS missing reason	BALANS timing (days poststroke)	BALANS trials (n)	COPvel-ml (mm/s)	COPvel-ml (mm/s)	WBA (%)	DCA (%)	GAIT included	GAIT missing reason		GAIT strides (n)	Gait speed (m/s)	SLA (ratio)	SLA direction
9	2	0	1	0	BBS-s < 1							0	FAC < 3					
9	2	4	2	0	technical issue	41						0	FAC < 3					
14	2	4	3	0	technical issue	61						1		1	11	0.372165529	1.040478952	non-paretic
14	2	4	5	1		92	2	12.2219502	11.30177809	0.396752413	0.59296074	1		1	13	0.339692786	1.040515454	non-paretic
14	2	0	0	0	BBS-s < 1							0	FAC < 3					
25	2	4	3	0	technical issue	41						0	unable to walk w/o assistance					
25	2	4	5	1		59	2	17.81760823	36.36646656	0.360810411	0.633574882	1		1	11	0.787146147	1.076570511	non-paretic
25	2	4	5	1		88	3	10.34700158	21.81366349	0.378331434	0.643473721	1		1	8	1.009178303	1.056634373	paretic
14	2	2	1	0	unable to stand barefooted w/o assistance							0	FAC < 3					
25	2	4	2	0	technical issue	39						0	FAC < 3					
19	2	4	4	1		59	2	7.769602624	11.63584273	0.336568821	0.586361234	0	refused to participate					
25	2	4	5	1		91	2	9.261768698	12.81110939	0.376301997	0.743730371	1		1	13	0.363278913	1.115907252	non-paretic
9	1	0	0	0	BBS-s < 1							0	FAC < 3					
14	2	1	1	1		41	2	22.34309416	29.07552652	0.347924162	0.878753878	0	FAC < 3					
19	2	3	2	1		62	3	26.63118327	24.7335246	0.284358262	0.652195083	0	FAC < 3					
25	2	3	3	1		89	2	24.62244336	23.62720765	0.400190441	0.916166999	0	unable to walk w/o assistance					
0	2	0	0	0	BBS-s < 1							0	FAC < 3					
0	2	3	1	1		38	2	22.4239543	24.97388055	0.408523844	1.406384546	0	FAC < 3					
9	2	4	2	1		57	3	25.49236314	25.26374806	0.463419087	1.368855262	0	FAC < 3					
14	2	4	3	1		91	3	26.40800308	28.13711543	0.447048593	1.330598736	1		1	8	0.274752392	1.137487834	non-paretic
0	1	0	0	0	BBS-s < 1							0	FAC < 3					
0	2	3	1	1		37	3	19.64060572	23.46828363	0.265272334	0.509339406	0	FAC < 3					
9	2	3	2	1		59	3	9.375819446	17.05141973	0.314641727	0.853500985	0	FAC < 3					
9	2	4	2	1		88	3	8.010554644	13.98017887	0.322930803	0.98158226	0	FAC < 3					
14	2	0	0	0	BBS-s < 1							0	FAC < 3					
14	2	4	3	1		36	3	17.0652884	17.2979275	0.426849012	0.860884035	0	unable to walk w/o assistance					
25	2	4	4	1		58	3	11.98596997	14.76235669	0.389215745	0.748421754	1		1	13	0.585000495	1.026188883	paretic
25	2	4	5	1		86	3	9.860799637	13.8112829	0.457548396	0.941480005	1		1	13	0.410283913	1.12196772	non-paretic
0	1	0	0	0	BBS-s < 1							0	FAC < 3					
0	2	3	2	1		40	2	24.80071186	21.80878569	0.196575351	1.6	0	FAC < 3					
14	2	3	2	1		60	3	20.10245393	15.19972487	0.242376494	1.6	0	FAC < 3					
14	2	3	2	1		87	3	25.448251	18.70320565	0.204902408	1.6	0	FAC < 3					
25	2	0	0	0	BBS-s < 1							0	FAC < 3					
33	2	4	3	1		37	3	10.46459013	23.22351936	0.616179086	-0.32398169	0	unable to walk w/o assistance					
25	2	4	5	1		57	3	11.6479785	19.92295412	0.616193822	0.006337394	1		1	10	0.842632741	1.027559583	non-paretic
25	2	4	5	1		84	3	8.887124297	15.4855153	0.538845179	-0.314962975	1		1	13	0.917539745	1.019290747	paretic
9	2	0	0	0	BBS-s < 1							0	FAC < 3					
19	2	3	1	1		40	3	30.91664411	27.959368	0.375282307	0.627171036	0	FAC < 3					
14	2	3	1	1		61	3	26.1133868	27.31311459	0.464012285	0.530235204	0	FAC < 3					
25	2	4	4	1		88	3	21.05910917	16.93444817	0.450299064	0.174637459	0	unable to walk w/o assistance					
0	1	0	0	0	BBS-s < 1							0	FAC < 3					
9	2	3	0	1		43	3	19.58934566	30.28392683	0.431240731	0.521897735	0	FAC < 3					
0	2	2	1	0	participants was unwell							0	FAC < 3					
9	2	3	1	1		87	3	22.90567595	26.91723474	0.405289829	0.731136369	0	FAC < 3					
25	2	0	1	0	BBS-s < 1							0	FAC < 3					
25	2	4	3	1		40	3	7.06026239	15.2193528	0.452799797	-0.247974188	5w not inclu		1				
25	2	4	4	1		57	3	6.574764097	12.33284935	0.406361949	0.229398653	1		1	14	0.616598287	1.024733169	non-paretic
25	2	4	5	1		84	3	5.446302481	11.17881389	0.430144941	0.20927076	1		1	11	0.882297034	1.054469854	non-paretic
0	2	1	0	0	unable to stand barefooted w/o assistance							0	FAC < 3					
14	2	4	2	1		36	3	10.49955108	12.38928475	0.240181183	1.6	0	FAC < 3					
14	2	4	3	1		57	3	7.498028674	11.03798081	0.351611519	0.518202445	1		1	12	0.556706829	1.089540261	non-paretic
14	2	4	4	1		93	3	6.502250806	9.731696704	0.323044437	0.438287393	1		1	17	0.719363959	1.013669412	non-paretic
14	2	0	0	0	BBS-s < 1							0	FAC < 3					
9	2	0	0	0	BBS-s < 1							0	FAC < 3					
14	2	2	1	1		58	3	32.86621088	24.41446123	0.437838193	0.546060014	0	FAC < 3					
14	2	2	1	1		85	3	30.68299927	18.00133525	0.390782554	0.00349145	0	FAC < 3					
14	2	0	0	0	BBS-s < 1							0	FAC < 3					
14	2	4	1	1		37	3	22.33591068	27.57826344	0.350253911	0.449423351	0	FAC < 3					
25	2	4	1	1		63	3	17.81473336	26.35866202	0.314650587	0.42485237	0	FAC < 3					
33	2	4	4	1		91	3	20.69044437	28.76681936	0.299782673	0.501629438	0	unable to walk w/o assistance					
19	0	0	0	0	BBS-s < 1							0	FAC < 3					
14	2	1	0	0	unable to stand barefooted w/o assistance							0	FAC < 3					
25	2	2	1	0	unable to stand barefooted w/o assistance							0	FAC < 3					
25	2	4	4	0	unable to stand barefooted w/o assistance							0	unable to walk w/o assistance					
25	2	0	0	0	BBS-s < 1							0	FAC < 3					
33	2	4	4	1		38	3	11.91212125	15.29366713	0.428586141	0.055807608	5w not inclu		1				
33	2	4	4	1		59	3	9.866527083	10.00996687	0.476760339	0.545027153	1		1	14	0.878464428	1.026615544	non-paretic
33	2	4	5	1		87	3	7.590821728	9.661722891	0.4983645	0.52285399	1		1	13	0.990727942	1.011404885	non-paretic
0	2	0	0	0	BBS-s < 1							0	FAC < 3					
14	2	4	2	1		36	3	19.47832389	23.68203125	0.386534015	0.700853895	0	FAC < 3					
14	2	4	3	1		57	3	15.07113577	23.14135343	0.360282128	0.733027748	0	unable to walk w/o assistance					
19	2	4	3	1		89	3	15.0199733	25.18680217	0.247404333	1.6	1		1	13	0.60539659	1.001874549	non-paretic
0	0	0	0	0	BBS-s < 1							0	FAC < 3					
9	2	2	1	0	laboratory unavailable							0	FAC < 3					
0	2	2	1	0	laboratory unavailable							0	FAC < 3					
0	2	2	1	0	laboratory unavailable							0	FAC < 3					
